# Three isoforms of the Atg16L1 protein contribute different autophagic properties

**DOI:** 10.1007/s11010-013-1616-8

**Published:** 2013-03-20

**Authors:** Tao Jiang, Beibei Qin, Jianqin He, Shuangyan Lin, Shiping Ding

**Affiliations:** The National Education Base for Basic Medical Sciences, School of Medicine, Zhejiang University, Hangzhou, Zhejiang China

**Keywords:** Autophagy, Atg16L1, Isoforms, Colocalisation, LC3

## Abstract

The mammalian Atg16L1 protein consists of a coiled-coil domain and a tryptophan-aspartic acid (WD) repeat domain and is involved in the process of autophagy. However, the mechanisms underlying the effect of the Atg16L1 isoforms on autophagy remain to be elucidated in humans. In the present study, we successfully cloned three isoforms: Atg16L1-1, which contains the complete sequence; Atg16L1-2, which lacks all of exon 8; and Atg16L1-3, which lacks the coiled-coil domain. Subsequent experiments showed that the three isoforms of Atg16L1 were colocalised with MDC within the cells. Quantitative analysis of fluorescence showed that the average number of dots of Atg16L1-1 that colocalised with MDC was higher than those of Atg16L1-2 and Atg16L1-3. The three isoforms of Atg16L1 also colocalised with the lysosome within the cells. The average number of dots of Atg16L1-1 that colocalised with the lysosome was higher than those of Atg16L1-2 and Atg16L1-3. However, although Atg16L1-1 and Atg16L1-3 colocalised with the mitochondria, Atg16L1-2 did not. Functional analysis showed that overexpression of the three isoforms of Atg16L1 had a stimulative effect on autophagy. Significant increase in the number of positive LC3-II dots per cell was observed in Atg16L1-1 (70.2 ± 2.39 dots); this number was greater than those of the other two isoforms. Atg16L1-2 appeared to have an average of 59.25 ± 2.22 LC3-II dots per cell. Atg16L1-3 appeared to have the least number of LC3-II dots per cell (48.25 ± 2.22 dots) (*P* < 0.001). Our results indicated that the degree of autophagy varied with different Atg16L1 isoforms. The different domains of Atg16L1 played different roles in the process of autophagy. The coiled-coil domain of Atg16L1 was involved in the process of autophagy.

## Introduction

Autophagy is a catabolic pathway conserved among eukaryotes [1]. Autophagy is initiated by the formation of the phagophore or isolated membrane, a crescent-shaped double membrane that expands and fuses to form a double-membrane vesicle, the autophagosome. Autophagosomes eventually fuse with lysosomes to degrade their contents [2, 3]. By this process, autophagy allows cells to rapidly eliminate large unwanted structures and recycle their energy, and it is most likely involved in restoring intracellular nutrients during starvation and as a quality control mechanism to protect the cell against damage caused by toxic macromolecules and damaged organelles [2, 4–6]. Furthermore, autophagy plays an important role in the destruction of some bacteria, such as *Mycobacterium tuberculosis*, *Salmonella typhimurium* and adherent invasive *Escherichia coli* [7].

The mammalian Atg16L1 protein contains an N-terminal Atg5-binding domain, a coiled-coil domain and a C-terminal WD-repeat domain. Mammalian Atg16L1 has been found to be much larger than yeast Atg16L1, which only contains an N-terminal Atg5-binding domain and a coiled-coil domain [8]. The N-terminal Atg5-binding domain and the coiled-coil domain can mediate homo-multimerisation and can interact with the Atg5–Atg12 conjugate [9, 10]. In addition, the WD repeats are protein interaction domains found in functionally diverse proteins, suggesting that there may be undiscovered binding partners of Atg16L1 that interact with this region [11, 12].

A series of studies have confirmed that Atg16L1 can form a complex with the Atg12–Atg5 conjugate and that together they are actively translocated to the phagophore and are further elongated during autophagosome formation [9, 13, 14]. Cadwell et al. have established two mouse lines (Atg16L1^HM1^ and Atg16L1^HM2^) in which Atg16L1 expression was disrupted by gene trap mutagenesis. Subsequent experiments in vitro and in vivo showed that Atg16L1^HM1^ and Atg16L1^HM2^ resulted in a lower LC3II/LC3I ratio and impaired autophagy adapter protein p62 in comparison to Atg16L1^WT^ [15]. Saitoh et al. reported that Atg16L1-deficiency disrupted the recruitment of the Atg12–Atg5 conjugate, but the wild type mouse embryonic fibroblasts did not exhibit impaired autophagy. These data implied that Atg16L1 was involved in the formation of the autophagosome [16–22].

Multiple isoforms of Atg16L1 exist as a result of alternative splicing events in humans [15, 23]. Zheng et al. have cloned the full-length cDNA of the human Atg16L1 protein, encoding 607 amino acids, by large-scale sequencing analysis of a human foetal brain cDNA library. Additionally, data from the EST database show that there might be at least four isoforms of Atg16L1. All of the four Atg16L1 isoforms contain a different domain [24].

These results have provided a structural basis for understanding the molecular structures and functions of Atg16L1 in autophagy. However, the effect of the Atg16L1 isoforms on autophagy in humans remains to be elucidated. In the present study, we successfully cloned three isoforms called Atg16L1-1, Atg16L1-2 and Atg16L1-3 with the aid of bioinformatics analysis. We aimed to further compare the function of these three Atg16L1 isoforms on autophagy.

## Materials and methods

### Bioinformatic analysis of the Atg16L1 isoforms

We searched for potential isoforms of Atg16L1 on the following websites: http://www.uniprot.org/Q676U5(A16L1_HUMAN) and http://www.ncbi.nlm.nih.gov/. The domain of the Atg16L1 protein was analysed using the following websites: http://pfam.sanger.ac.uk/; http://bioinf.cs.ucl.ac.uk/ and http://www.rcsb.org/pdb. The coiled-coil domain of the Atg16L1 isoforms was analysed with the Swiss-PdbViewer 4.0.4 software.

### Reverse transcriptase (RT)-PCR

Total RNA was isolated from HeLa cells using RNAiso Plus (TaKaRa), and cDNA was synthesised from mRNA using the PrimeScript II 1st Strand cDNA Synthesis Kit (TaKaRa). Atg16L1-1, Atg16L1-2, Atg16L1-3 and β-actin PCR products were produced using the following oligonucleotide primers:

Atg16L1-1:

F1: 5′-CTCGAGATGTCGTCGGGCCTCCGCGCCGCTGACTT-3′,

R1: 5′-GAATTCTCAGTACTGTGCCCACAGCACAGCTTTGC-3′;

Atg16L1-2:

F2: 5′-CTCGAGATGTCGTCGGGCCTCCGCGCCGCTGACTT-3′,

R2: 5′-GAATTCTCAGTACTGTGCCCACAGCACAGCTTTGC-3′;

Atg16L1-3:

F3: 5′-CTCGAGATGCAGCGGAAGGACAGGGA-3′,

R3: 5′-GAATTCTCAGTACTGTGCCCACAGCACAGCTTTGC-3′;

β-actin:

F4: 5′-CTGGGACGACATGGAGAAAA-3′,

R4: 5′-AAGGAAGGCTGGAAGAGTGC-3′.

### Plasmid construction

The three isoforms of human Atg16L1 were cloned into the multiple cloning site of pEGFP-C1 to generate pEGFP–Atg16L1-1, pEGFP–Atg16L1-2 and pEGFP–Atg16L1-3. Using the manufacturer’s protocol, pcDNA3.1(+)–Atg16L1-1, pcDNA3.1(+)–Atg16L1-2 and pcDNA3.1(+)-Atg16L1-3 were also successfully constructed.

### Cell culture

HeLa cells were grown in DMEM (Sigma) supplemented with 10 % foetal bovine serum (Gibco), 2 mM l-glutamine and appropriate antibiotics in a 5 % CO_2_ incubator at 37 °C.

### Fluorescence detection of monodansylcadaverine (MDC), the lysosome and the mitochondrion

Before transfection, cells were plated into 24-well plates and incubated in a 5 % CO_2_ incubator at 37 °C. Transient transfection of pEGFP, pEGFP–Atg16L1-1, pEGFP–Atg16L1-2 and pEGFP–Atg16L1-3 was carried out using the Attractene transfection reagent (Qiagen) according to the manufacturer’s protocol. After 48 h incubation, the cell culture medium was replaced with new culture medium that contained MDC (Sigma), LysoTracker (Invitrogen) or MitoTracker Red CM-H_2_Xros (Invitrogen) for 30 min, for the purpose of staining. Cells cultured on coverslips were washed with PBS and fixed with 4 % paraformaldehyde. Samples were examined under a fluorescence laser scanning confocal microscope (BX61W1-FV1000, Olympus, Tokyo, Japan). Related quantitative parameters were analysed with Image-Pro Plus 6.0 software.

### Fluorescence detection of LC3

Cotransfection of pEGFP–LC3 with pcDNA3.1(+), pcDNA3.1(+)–Atg16L1-1, pcDNA3.1(+)–Atg16L1-2 and pcDNA3.1(+)–Atg16L1-3 was carried out using the Attractene transfection reagent (Qiagen) according to the manufacturer’s protocol. After 48 h incubation, the cotransfected cells were stimulated with rapamycin for a further 12 h, at which time the cells were treated as described above. The samples were observed under a fluorescence laser scanning confocal microscope, and the GFP–LC3 positive dots per cell were counted.

### Statistical analysis

For in vitro studies, individual transfections were treated as independent experiments, and unpaired Student’s *t* tests were performed on the treatment versus the non-treatment groups. All in vitro studies were performed in triplicate and repeated on three independent occasions.

## Results

### The structure of Atg16L1 isoforms

The Atg16L1 protein consists of two domains: a coiled-coil domain, located at the N-terminal region, which can form a homo-oligomer with Atg5 and Atg12, and seven copies of the WD repeats which located at the C-terminal region in the form of a β-transducin repeat, which is a short structural motif of approximately 40 amino acids terminating in a tryptophan-aspartic acid (WD) dipeptide.

**Fig. 1:**
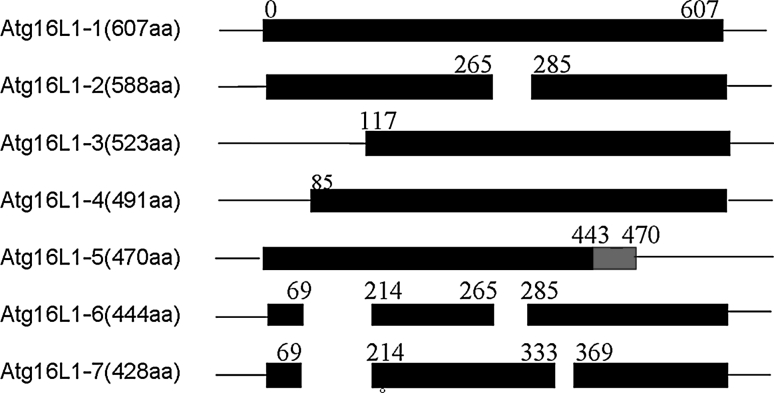
Schematic presentation of seven isoforms of human Atg16L1. The first was symbol of the wide type of human Atg16L1 containing complete length of sequences which encoded a protein of 607 amino acid residues, the other *six bars* were shown isoforms of Atg16L1 with specific amino acid residues, respectively

Data from the Genomic DNA database, EST database, NCBI database and UniProtKB database showed that seven isoforms of Atg16L1 exist in *Homo sapiens*. Figure 1 shows that Atg16L1-1 contains the complete sequence encoding a protein of 607 amino acid residues. Atg16L1-2 lacks all of exon 8 (57 bp). Atg16L1-3 and Atg16L1-4 encode proteins lacking 116 and 84 amino acid residues at the N-terminus, respectively. As for Atg16L1-5, the region of the amino acid residues from position 443 to position 470 has been changed in comparison with the canonical sequence and lacks part of the WD repeats at the C-terminus (amino acid residues at positions 471–607). Atg16L1-6 encodes a protein missing amino acid residues at positions 70–213 and also 266–284. Similarly, Atg16L1-7 lacks amino acid residues at positions 70–213 and positions 334–368.

**Fig. 2:**
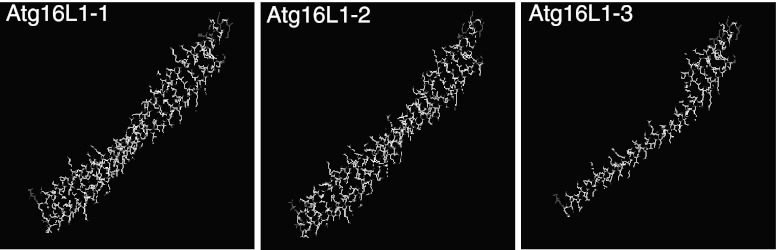
Three-dimensional analysis of the coiled-coil domain for Atg16L1-1, Atg16L1-2 and Atg16L1-3. It was shown that Atg16L1-1 had a similar conformation to Atg16L1-2. However, Atg16L1-3 had a different conformation from the other two isoforms because of lacking 116 amino acids at the N-teminal region

The RT-PCR analysis showed that two isoforms (Atg16L1-1 and Atg16L1-2) were expressed in HeLa cells and encoded 607 amino acids and 588 amino acids, respectively (data not shown). Atg16L1-3 was also cloned. Three-dimensional analysis of the coiled-coil domain showed that Atg16L1-1 has a conformation which is similar to that of Atg16L1-2. However, Atg16L1-3 has a different conformation because of the lack of 116 amino acids at the N-terminal region (Fig. 2). For this reason, we hypothesised that the three isoforms of Atg16L1 might have different effects on autophagy.

### Colocalisation of the Atg16L1 isoforms with MDC

**Fig. 3:**
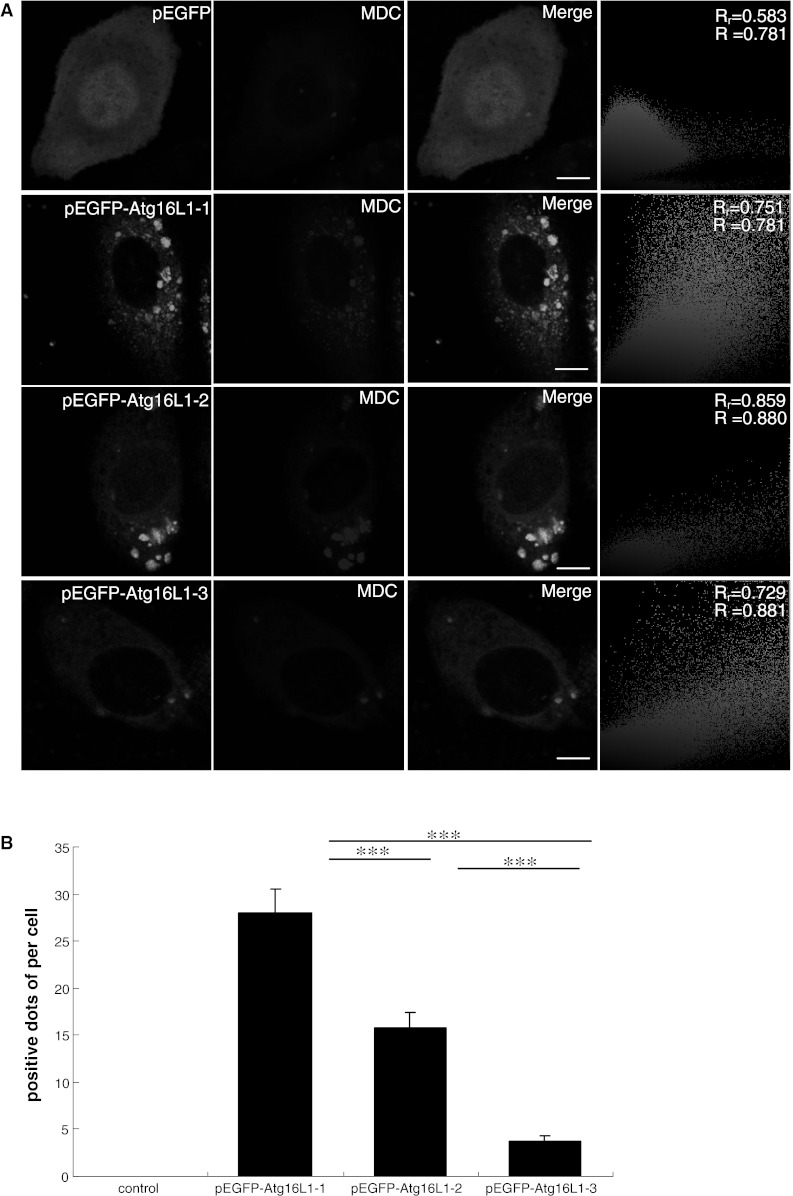
Three isoforms of Atg16L1 were colocalised with autophagic vacuoles labelled by MDC. **a** HeLa cells expressing pEGFP, pEGFP–Atg16L1-1, pEGFP–Atg16L1-2 and pEGFP–Atg16L1-3 for 48 h were labelled by 50 μM MDC for 10 min and fixed with 4 % paraformaldehyde for 15 min before imaging in confocal fluorescence microscopy. Fluorescence intensity analysis of multicolour confocal immunofluorescence microscopy images for pEGFP, pEGFP–Atg16L1-1, pEGFP–Atg16L1-2, pEGFP–Atg16L1-3 in autophagic vacuole labelled by monodansylcadaverine. *Scale bars* 10 μm. **b** Positive dots of quantitative fluorescence intensity analysis of multicolour confocal immunofluorescence microscopy images for pEGFP, pEGFP–Atg16L1-1, pEGFP–Atg16L1-2 and pEGFP–Atg16L1-3 in autophagic vacuole labelled by MDC. Cells treated as indicated above were incubated with 50 μM MDC for 10 min at 37 °C. The positive dots were measured by fluorescence photometry as indicated in experimental procedures. Data were represented as mean ± SEM; ***P < 0.001

MDC can accumulate in the acidic sub-cellular compartments in the autophagy process, especially in the later stages [25]. Therefore, it can be generally considered to be a specific marker for autophagic vacuoles in vitro and in vivo [26]. To investigate whether Atg16L1-1, Atg16L1-2 and Atg16L1-3 participated in the autophagic pathway, MDC fluorescence experiments were performed. Figure 3a showed that three isoforms of Atg16L1 were colocalised with MDC. Quantitative analysis of fluorescence showed that the average number of dots of the Atg16L1-1 isoform observed that colocalised with MDC was 28.0 ± 2.58, which was higher than those of Atg16L1-2 and Atg16L1-3, at 15.75 ± 1.71 and 3.75 ± 0.75, respectively (*P* < 0.001) (Fig. 3b). These results showed that the degree of colocalisation varied significantly among these three isoforms in HeLa cells.

### Colocalisation of the Atg16L1 isoforms with the lysosome

**Fig. 4:**
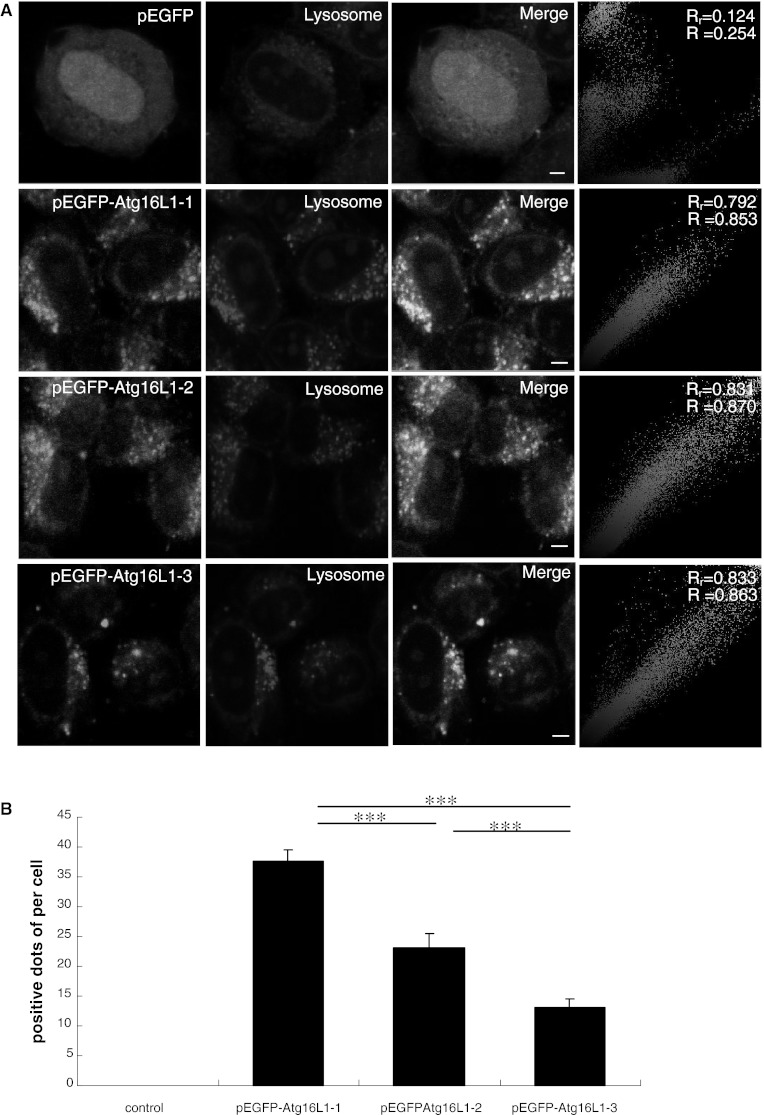
Colocalisation of pEGFP, pEGFP–Atg16L1-1, pEGFP–Atg16L1-2 and pEGFP–Atg16L1-3 in lysosome. **a** HeLa cells were transiently transfected with pEGFP, pEGFP–Atg16L1-1, pEGFP–Atg16L1-2 and pEGFP–Atg16L1-3. After 48 h, cells were incubated with Lyso-Tracker for 2 h. Cells were washed with PBS and immediately analysed by fluorescence microscopy. pEGFP–Atg16L1 are displayed in *green* and Lyso-Tracker are in *red*. Colour colocalisation Images of pEGFP, pEGFP–Atg16L1-1, pEGFP–Atg16L1-2, pEGFP–Atg16L1-3 in lysosome. *Scale bars* 10 μm. (B) Positive dots of quantitative fluorescence intensity analysis of multicolour confocal immunofluorescence microscopy images for pEGFP, pEGFP–Atg16L1-1, pEGFP–Atg16L1-2 and pEGFP–Atg16L1-3 in lysosome. Data were represented as mean ± SEM; ***P < 0.001. (Color figure online)

In the later stage of autophagy, autophagosomes fuse with lysosomes to form an autophagolysosome in which some unwanted components have been degraded [27]. Figure 4a showed that the three isoforms of Atg16L1 were colocalised with lysosomes that were involved in autophagy. Quantitative analysis of fluorescence indicated that It existed a difference among the three isoforms of Atg16L1. The average number of dots of the isoform Atg16L1-1 observed that colocalised with the lysosome was 37.67 ± 1.75, which was higher than those of Atg16L1-2 and Atg16L1-3, at 23.14 ± 2.27 and 13.0 ± 1.58, respectively (*P* < 0.001) (Fig. 4b).

### Colocalisation of the Atg16L1 isoforms with the mitochondria

**Fig. 5:**
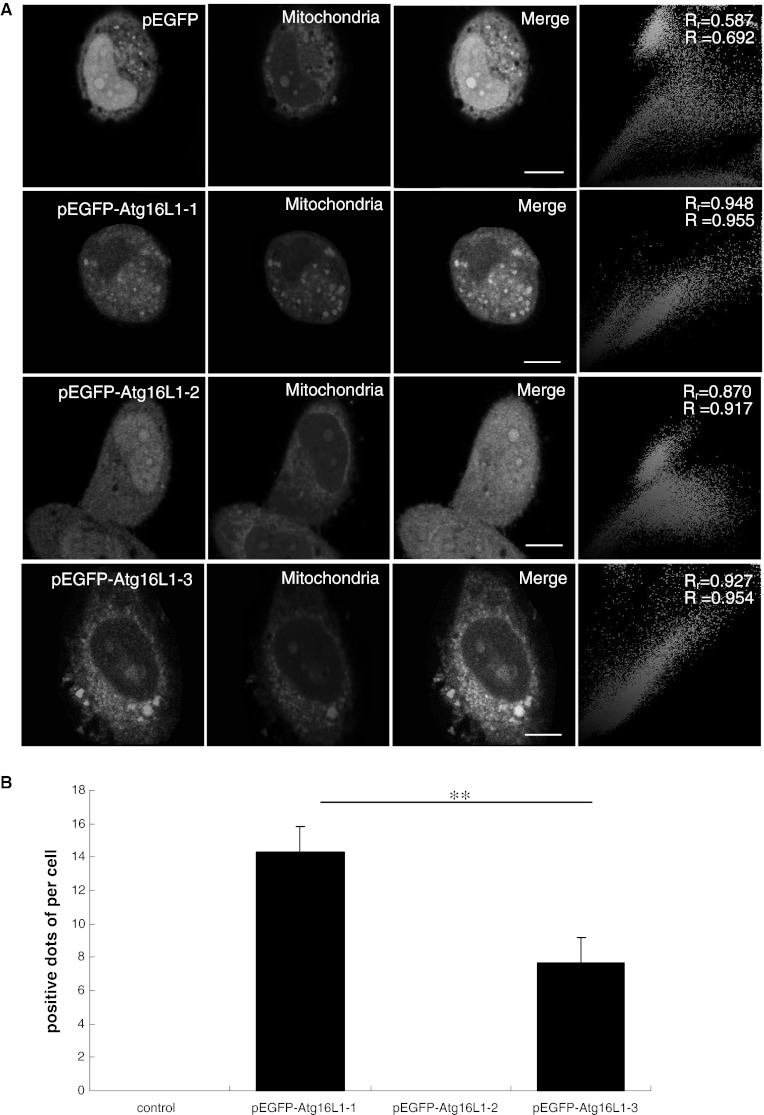
Colocalisation of pEGFP, pEGFP–Atg16L1-1, pEGFP–Atg16L1-2 and pEGFP–Atg16L1-3 in mitochondria. **a** HeLa cells transfected with plasmids encoding pEGFP, pEGFP–Atg16L1-1, pEGFP–Atg16L1-2 and pEGFP–Atg16L1-3 were stained with Mito-Tracker and imaged by live-cell confocal microscopy. Colour colocalisation images of pEGFP, pEGFP–Atg16L1-1, pEGFP–Atg16L1-2 and pEGFP–Atg16L1-3 in mitochondria. *Scale bars* 10 μm. **b** Positive dots of quantitative fluorescence intensity analysis of multicolour confocal immunofluorescence microscopy images for pEGFP, pEGFP–Atg16L1-1, pEGFP–Atg16L1-2 and pEGFP–Atg16L1-3 in mitochondria. Data were represented as mean ± SEM; **P < 0.01

Mitochondria are also involved in the process of autophagy. Unexpectedly, Atg16L1-1 and Atg16L1-3 were colocalised with mitochondria, but Atg16L1-2 was not (Fig. 5a). Quantitative analysis of fluorescence indicated that the number of positive dots observed for the isoform Atg16L1-1 was 14.33 ± 1.53 per cell, which was more than that observed for Atg16L1-3 (7.67 ± 1.53 per cell) (*P* < 0.01) (Fig. 5b).

### Functional effects of Atg16L1 isoforms on autophagy

**Fig. 6:**
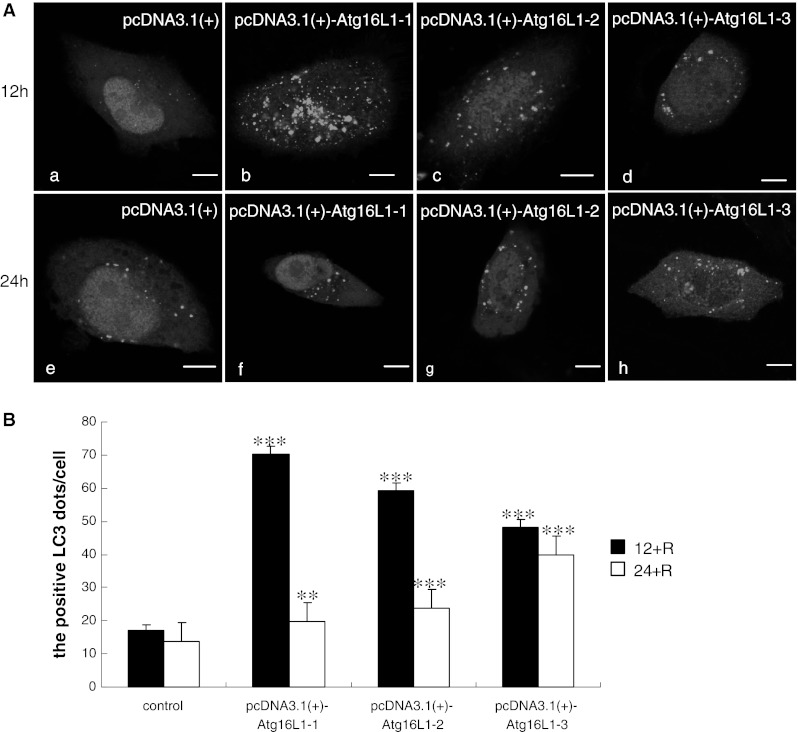
Confocal fluorescence microscopy of Atg16L1 wild-type and isoforms cotransfected HeLa cells with GFP–LC3 (*green*) with rapamycin induction for 12 and 24 h. **a** HeLa cells were cotransfected with pEGFP–LC3 and pcDNA3.1(+), or pcDNA3.1(+)–Atg16L1 as described in “Materials and methods” section. After 48 h incubation, the cotransfected cells were induced with rapamycin for further 12 h (**a**–**d**) and 24 h (**e**–**h**), respectively, and were imaged by microscopy. *Scale bars* 10 μm. **b** Quantification of dot-shaped GFP–LC3 signals in HeLa cells stimulated as described above. Data were represented as mean ± SEM; **P < 0.01 versus control, ***P < 0.001 versus control. (Color figure online)

LC3 is a mammalian homolog of the yeast Atg8 [28]. It has been shown that LC3 exists in two forms: the 18 kDa cytosolic form (LC3-I) and the 16 kDa processed form (LC3-II), which is located on the autophagosomal membrane [29]. A number of studies have suggested that tracking the conversion of LC3-I to LC3-II is indicative of autophagic activity [7]. To better characterise the effect of Atg16L1 isoforms on autophagy, we cotransfected pEGFP–LC3 and pcDNA3.1(+), pcDNA3.1(+)–Atg16L1-1, pcDNA3.1(+)–Atg16L1-2, pcDNA3.1(+)–Atg16L1-3 into HeLa cells and incubated with rapamycin for 12 h. Figure 6a showed that the cells expressing each of the three Atg16L1 isoforms expressed higher levels of LC3-II than the control cells, which implied that overexpression of the three isoforms of Atg16L1 had a stimulatory effect on autophagy. Significant increase in the number of positive LC3-II dots per cell was observed in the wild type Atg16L1-1 isoform (70.2 ± 2.39 dots), which exhibited more dots than the other two isoforms. Atg16L1-2 appeared to have an average of 59.25 ± 2.22 LC3-II dots per cell. Atg16L1-3 appeared to have the least number of LC3-II dots per cell (48.25 ± 2.22 dots) (*P* < 0.001) (Fig. 6b). These results suggested that the three isoforms of Atg16L1 varied in their effect on autophagy.

To further confirm the precise function of Atg16L1 in autophagy, the transfected cells were incubated with rapamycin for 24 h (Fig. 6a). Figure 6b shows that the numbers of LC3-II dots per cell were 19.75 ± 1.53 (*P* < 0.01), 23.75 ± 1.50 and 40 ± 1.0 (*P* < 0.001) in the three groups, respectively. Compared with the number observed for 12 h, the number of LC3-II dots for 24 h was reduced significantly. The phagophore began to fuse with the lysosome and further degraded its contents in a later stage of autophagy inside the cell. However, it should be noted that for isoform Atg16L1-3, LC3-II decreased slowly, which implied that the absence of the coiled-coil domain might have an inhibitory effect on the process of autophagy.

## Discussion

In the present study, we performed experiments to study the effects of Atg16L1 isoforms on autophagy. The degree of autophagy varied with different Atg16L1 isoforms.

LC3II/LC3I has been considered to be a quantitative index of autophagy in the classical autophagy pathway [30]. Our results showed that when the isoforms of Atg16L1 were overexpressed, the number of positive LC3-II dots increased. These data implied that over-expression of the Atg16L1 protein had an effect on autophagy and could induce the formation of autophagic vacuoles. Moreau et al. also supported our view [31]. In contrast to our view, Fujita et al. reported that overexpression of Atg16L1 inhibited autophagosome formation in PC12 cells [11]. Saitoh et al. reported that overexpression of the Atg16L1 protein had no influence on autophagy in wild type mouse embryonic fibroblasts [8]. This discrepancy between our findings and published data may be due to differences in experimental design or to differences among cell types. However, whether exogenous and endogenous Atg16L1 proteins have a similar effect on autophagy still remains to be verified.

Recent studies indicated that the coiled-coil domain of Atg16L1 was the binding site with the Atg12–Atg5 conjugate, which is required for autophagy [8, 9, 15, 32]. Structure-based mutational analyses of the coiled-coil domain of the Atg16L1 protein indicated that there existed some conserved surface amino acid residues that are necessary for autophagy [28]. Ravikumar et al. (2010) found that over-expression of a truncated Atg16L1 protein lacking the coiled-coil domain in HeLa cells resulted in very few autophagic vesicles and that these vesicles were unable to envelop cholera toxin [33]. Our results showed that the degree of colocalisation of the Atg16L1 isoforms with the lysosome, MDC and the mitochondria varied between Atg16L1-1 and Atg16L1-3. Their effects on autophagy were different. Compared with Atg16L1-1, Atg16L1-3 appeared to have less localisation with the sub-cellular organelle and to have the least number of positive LC3-II dots in the cell. These data demonstrated that the coiled-coil domain of Atg16L1 was involved in the process of autophagy.

Effective autophagy is dependent on the equilibrium between the formation and elimination of autophagosomes, and a deficit in any part of the stepwise maturation process will cause autophagic dysfunction [7]. Atg16L1-3 had no coiled-coil domain, and when Atg16L1-3 was overexpressed, the formation of LC3-II dots decreased more slowly with time (12 and 24 h) in comparison with Atg16L1-1. These data implied that the process of maturation from the autophagosome into the autolysosome might be blocked. Based on these results, we hypothesised that Atg16L1 was not only involved in the recruitment of the Atg12–Atg5 conjugate to the isolation membrane but also participated in the maturation of the autophagosome into the autolysosome through its coiled-coil domain.

Furthermore, our results showed that Atg16L1-2 had no exon 8. Functional experiments demonstrated that Atg16L1-2 had a moderate effect on autophagy. This finding might be because exon 8 deficiency in Atg16L1 had an influence on its structure. This result was then confirmed by Mizushima N’s data regarding Atg16Lα, a variant lacking all of exons 8 and 9, which was transfected into HeLa cells and resulted in increased levels of p62 associated with autophagy [9]. However, the precise mechanism of Atg16L1-2 with respect to autophagy remains to be further elucidated.

In conclusion, the present study suggested that the degree of autophagy varied with Atg16L1 isoforms. The different domains of Atg16L1 played different roles in the process of autophagy.
